# Latent Factor Structure of Dynamic Postural Control and Ankle Mobility in Young Female Volleyball Players During Single-Leg Tasks: A Pilot Study

**DOI:** 10.3390/jfmk11030264

**Published:** 2026-07-01

**Authors:** Koichi Moriguchi, Kuniaki Moridera, Tomoki Noguchi, Nariyuki Mura, Toshiaki Sato, Hiroshi Katoh

**Affiliations:** 1 Department of Rehabilitation, Moridera Orthopedic, Fukuoka 850-0068, Japan; 2 Graduate School of Health Sciences, Yamagata Prefectural University of Health Sciences, Yamagata 990-2212, Japan; nmura@yachts.ac.jp (N.M.); tsato@yachts.ac.jp (T.S.); 3 Department of Orthopedic Surgery, Moridera Orthopedic, Fukuoka 850-0068, Japan; k@moridera-med.jp; 4 Department of Rehabilitation, Ishiko Orthopedic Clinic, Fukuoka 820-0068, Japan; tomo.noguchi27@gmail.com

**Keywords:** postural balance, athletes, volleyball, stabilogram diffusion analysis, modified Star Excursion Balance Test

## Abstract

**Background:** This study aimed to explore the relationships among different indices and the underlying latent structure of dynamic postural control in young female volleyball players. It used factor analysis of indices from stabilogram diffusion analysis (SDA), the modified Star Excursion Balance Test (mSEBT), and the Weight-Bearing Lunge Test (WBLT) to generate hypotheses regarding the relationships among these measures. **Methods:** In total, 34 female middle- and high-school volleyball players participated in this study. The SDA was performed using center-of-pressure (COP) data obtained during a single-leg vertical jump landing task, and the critical point (CP) was calculated. Lower-limb reach distances in the anterior (ANT), posteromedial (PM), and posterolateral (PL) directions were measured using the modified mSEBT. In addition, hallux-to-wall distance (HWD) was measured using the WBLT. Exploratory factor analysis was conducted to examine the latent factor structure among these indices. **Results:** The Kaiser–Meyer–Olkin value was 0.63, and Bartlett’s test of sphericity had significant results (*p* < 0.001). Considering the factor retention decision and the study’s theoretical framework, a two-factor solution was adopted for factor analysis, yielding a cumulative explained variance of 79.77%. Factor 1 had high factor loadings for the ANT, PM, and PL directions. Meanwhile, factor 2 showed high factor loadings for CP and HWD. **Conclusions:** Factor 1 reflected the spatial dynamic balance ability associated with the mSEBT and may be related to it. Moreover, factor 2 may indicate the temporal characteristics of COP variability assessed via the SDA and based on ankle mobility-related characteristics. However, because this was an exploratory pilot study with a small sample size, the findings should be considered as preliminary hypotheses.

## 1. Introduction

Several sports movements, such as jump landing and changing direction, require advanced dynamic postural control [1,2,3,4,5,6]. In movements involving single-leg support, sensory input, muscle output, and neural control strategies must function in an integrated manner to appropriately control the body’s center of mass in response to perturbations [7,8]. In volleyball, movements involving single-leg support, such as landing after spiking or blocking, are commonly required [3,4]. In particular, young female players exhibit differences in postural control during landing movements due to neuromuscular control characteristics and lower-limb alignment associated with growth and development [9]. Considering these sport-specific characteristics, the dynamic postural control characteristics of young female volleyball players should be evaluated from multiple perspectives.

The Star Excursion Balance Test (SEBT) and the modified SEBT (mSEBT) are widely used to evaluate dynamic postural control [10,11,12,13,14,15]. The mSEBT is used to assess dynamic balance ability and reach distance in three directions, serving as an indicator of these abilities. However, the mSEBT is influenced by multiple factors, including ankle range of motion, muscle strength, proprioception, and trunk control [16,17,18].

**Figure 1:**
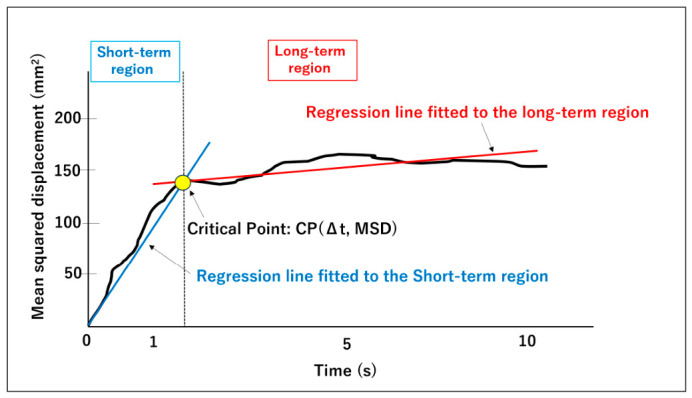
SDA analyzes COP time-series data. A schematic of a typical stabilogram diffusion curve, based on the mean square displacement (MSD) from the COP time series, shows two regions: a short-time region and a long-time region. The intersection of the regression lines fitted to each region marks the critical point (CP). The short-time region indicates open-loop control without sensory feedback, while the long-time region reflects closed-loop control with sensory feedback. Hence, the CP signifies the transition from open-loop to feedback control.

In recent years, postural control has increasingly been viewed as a complex system, and the importance of nonlinear analyses of postural sway data obtained from force plates, motion capture systems, and accelerometers has been emphasized [19]. Accordingly, attention has shifted toward evaluating not only the spatial aspects of dynamic balance ability but also the temporal characteristics of postural sway. Stabilogram diffusion analysis (SDA), which uses time-series data from the center of pressure (COP), is used to analyze temporal changes in postural control. SDA is a technique for examining the time-related features of COP variability. Its key parameter, the critical point (CP), has been proposed as an indicator of the temporal transition in COP fluctuation patterns (Figure 1). Consequently, SDA has been used to evaluate the temporal aspects of postural control, which differ from the spatial dynamic balance assessed by the mSEBT [20,21].

The mSEBT reflects spatial dynamic balance ability, and SDA can serve as an indicator of the effects of temporal neural control strategies. Thus, these measures may evaluate different aspects of dynamic postural control. Further, dynamic postural control is likely influenced not only by spatial dynamic balance ability and temporal neural control characteristics but also by structural constraints related to ankle function. In particular, ankle dorsiflexion range of motion is involved in controlling anterior tibial inclination and center-of-mass displacement during landing. It may also be involved in spatial balance ability and temporal postural control strategies [22,23,24]. Ankle mobility has been linked to COP sway and postural stability, and changes in ankle function can influence COP control during postural regulation [25,26]. Thus, ankle dorsiflexion range of motion may be associated with not only to the spatial dynamic balance evaluated by the mSEBT but also to the temporal aspects of COP variability measured by the SDA. However, dynamic postural control is a complex process involving neuromuscular control, muscle strength, activation patterns, sensory input, and cognitive function [27,28], making it challenging to fully assess with only a few measures such as the mSEBT, SDA, and ankle mobility. Therefore, reach distances from the mSEBT, CP from the SDA, and ankle dorsiflexion range of motion should be viewed as indicators of specific facets of dynamic postural control. However, the latent structure underlying the associations among these indicators has not yet been completely elucidated.

This study aimed to analyze the relationships among various postural control measures obtained from the mSEBT, SDA, and ankle mobility assessments in young female volleyball players. Using factor analysis, we explored their underlying factor structure and generated hypotheses. We expected that the mSEBT and SDA would each form separate latent factors, representing spatial dynamic balance and postural control, respectively. Meanwhile, ankle dorsiflexion range of motion may be linked to both the mSEBT and SDA indicators, as it is a key physical function of dynamic postural control. However, its exact role within the latent structure was examined without prior assumptions.

## 2. Methods

### 2.1. Study Design

This was an exploratory cross-sectional study.

### 2.2. Participants

**Figure 2:**
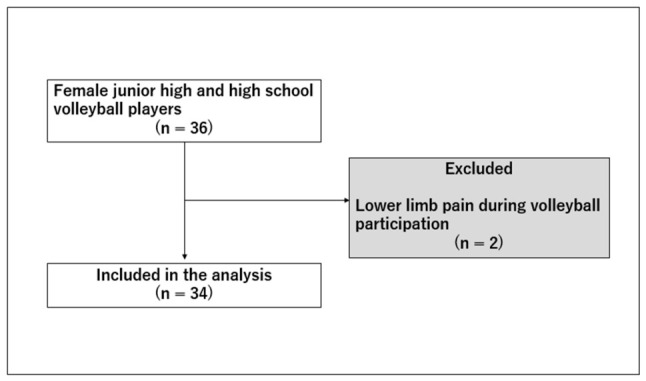
Flowchart of the inclusion and exclusion of participants.

The study included 34 female volleyball players from one junior high school and two high school teams who consented to participate (Table 1). The inclusion criteria were as follows: (1) students who participated in club activities without any restrictions, (2) those without any history of psychiatric disorders, (3) those without any history of neurological disorders, (4) those without any history of lower-limb orthopedic surgery within 1 year before the assessment, and (5) those without any history of lower-limb injury requiring absence from sports participation for >2 weeks within the last 6 months. The exclusion criteria included individuals with lower-limb pain during sports participation. Additionally, a pre-participation interview confirmed that none of the participants had a history of chronic ankle instability (CAI) or recurrent ankle sprains (Figure 2). After obtaining the coaches’ cooperation, the study’s purpose and procedures were explained to both the players and their parents. Written informed consent was then obtained from all participants and their parents. This study was approved by the institutional ethics committee of our hospital (Approval No. 25–001).

### 2.3. Tasks

The tasks comprised a landing-stabilization task following a single-leg vertical jump and the mSEBT task.

### 2.4. Measurement Procedures

Measurements were obtained using a force plate (SS-FP40AO; Sports Sensing Co., Ltd., Fukuoka, Japan) and a tape measure (Shinwa Measuring Co., Ltd., Niigata, Japan).

The outcomes of dynamic posture control were the SDA parameters derived from COP data obtained during the single-leg vertical jump landing test and the modified SEBT (mSEBT). In addition, ankle joint mobility was assessed using the Weight–Bearing Lunge Test (WBLT) [29].

The mSEBT is a common test used for evaluating lower-limb dynamic balance, with reliability scores reported to range from ICC = 0.51 to 0.95. It has also been linked to muscle strength, joint range of motion, proprioception, and neuromuscular control [30,31]. The WBLT shows high intra-rater reliability (0.97–0.98) and inter-rater reliability (0.99) for measuring ankle dorsiflexion range of motion [32]. SDA analyzes the temporal structure of postural sway from COP time-series data [33], and its reliability and applicability depend on measurement conditions like the number of trials and their duration [21]. Additionally, SDA reliability varies with participant characteristics, including visual conditions, age, and disease status [34]. Consequently, it is frequently used to characterize the temporal features of postural control.

The force plate sampling frequency was chosen as 1 kHz after a preliminary analysis comparing 500 Hz, 1 kHz, and 2 kHz. The results showed no substantial differences in CP between 1 kHz and 2 kHz, but the 500 Hz condition exhibited higher variability in estimated CP values. Thus, a 1 kHz sampling rate was selected for this study to ensure consistent CP estimation. All measurements were performed on the stance leg, which is the nondominant leg that plays a primary role in balance control and postural stability [35]. In this study, the dominant leg was identified, and the contralateral leg was considered the stance leg. In volleyball, asymmetrical movements occur during spiking and landing, with the nondominant leg serving as both the support and landing leg [36]. Therefore, this study focused on the nondominant leg, as it was deemed more suitable for evaluation during a single-leg support task. The dominant leg was determined based on the item “Which foot do you kick a ball with?” from the Waterloo Footedness Questionnaire-Revised [37]. This item has shown 100% agreement with observational assessments [38].

#### 2.4.1. Single-Leg Vertical Jump Landing Test

**Figure 3:**
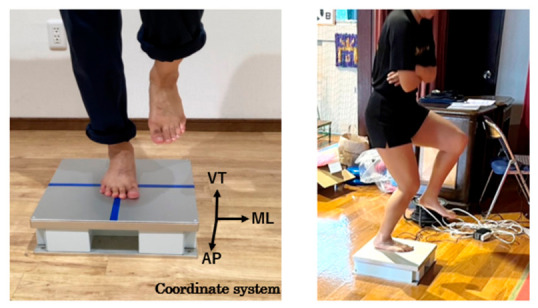
Single-leg vertical jump landing test.

The participants were instructed to maintain a single-leg standing position on the force plate with both arms crossed in front of the chest and eyes open and fixed on a visual marker placed 3 m in front of them. The supporting foot was positioned such that the center of the calcaneus and the midpoint between the second and third toes were aligned with a line along the anteroposterior axis passing through the center of the force plate. The participants were instructed to perform a vertical jump as high as possible from a single-leg stance on the pivot foot, land on the same lower limb, and maintain a stable standing position for 20 s. A trial was considered invalid and repeated if the participant could not maintain the single-leg stance for 20 s or if compensatory balance strategies using the upper limbs were observed. The number of trials was not predetermined; the task was repeated until a valid trial was obtained, and the first valid trial was used for analysis. In this study, 28 participants succeeded on the first attempt, 5 on the second, and 1 on the third (Figure 3).

#### 2.4.2. mSEBT

**Figure 4:**
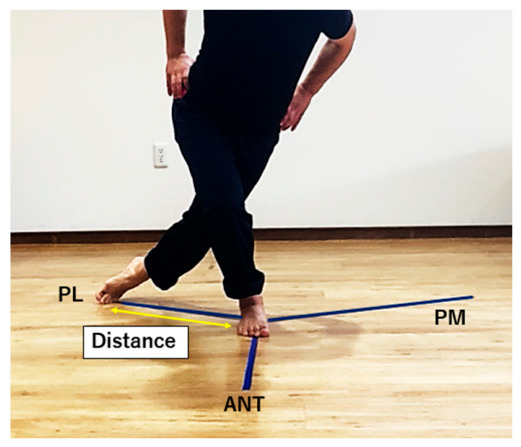
mSEBT.

The participants were instructed to place both hands on their hips and maintain a single-leg stance on the test limb while reaching as far as possible with the nonstance limb in the ANT, PM, and PL directions (Figure 4) [12]. Regarding the learning effect in the mSEBT, reach distance stabilizes after approximately six trials [13,14]. Therefore, the participants in this study performed six practice trials in each direction before data collection. A trial was considered invalid and repeated if the participant did not maintain a single-leg stance, either hand left the waist, the heel of the stance limb lost contact with the floor, or the nonstance limb touched the floor during the reach.

#### 2.4.3. WBLT

**Figure 5:**
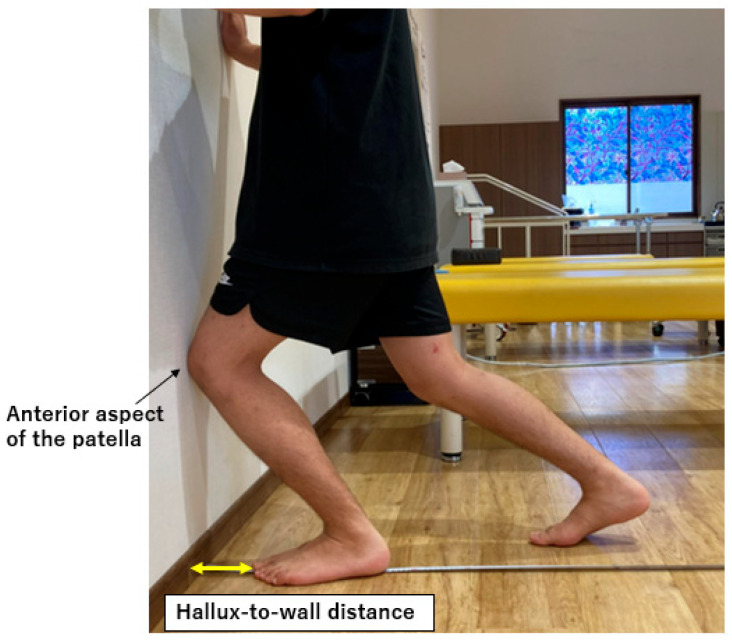
WBLT.

The stance limb was positioned such that the line connecting the center of the calcaneus and the lateral femoral condyle was perpendicular to the floor, with the heel in contact with the floor considered as the starting position. With the heel maintained in contact with the floor, the knee was flexed until the anterior aspect of the patella contacted the wall, indicating maximal ankle dorsiflexion. The hallux-to-wall distance (HWD) was measured with a tape measure (Figure 5). Before this study, a preliminary test with five participants showed that the intra-rater reliability of the WBLT was ICC(1,3) = 0.96, and the standard error of measurement was 0.68 cm.

### 2.5. Data Analysis

The outcome measures included the CP(s) derived from the SDA, calculated from COP data obtained during the single-leg vertical jump landing test; normalized reach distances (%) in the ANT, PM, and PL directions, measured using the mSEBT; and normalized HWD (%) measured with the WBLT.

#### 2.5.1. SDA Using the COP Data Obtained During the Single-Leg Vertical Jump Landing Test

The SDA conceptualizes COP data as a stochastic process and analyzes postural control using random-walk theory. The MSD for each time interval (*Δt*) was calculated as follows [33]:

$$\langle ∆r^{2}(m)\rangle =\frac{1}{N-m}\sum_{i=1}^{N-m}[{\left(x_{i+m}-x_{i}\right)}^{2}+{\left(y_{i+m}-y_{i}\right)}^{2}]$$

where *x_i_* and *y_i_* represent the COP coordinates at time point *i*, *N* denotes the total number of data points, and m represents the number of data points corresponding to the time interval *Δt*. In SDA, a time lag of roughly 0.01–1.0 s is typically chosen, based on the sampling frequency, to generate the MSD curve [33]. In our study, *Δt* was set to 0.1 s following prior research [21,39]. Linear regression analysis using the least squares method was performed on the resulting MSD curve. In addition, the CP, the time at which the transition from the short-term region to the long-term region occurs, was assessed. Although SDA allows for the calculation of multiple indices, this was an exploratory pilot study with a limited sample size; therefore, to account for the number of variables included in the factor analysis, only CP, which represents the temporal characteristics of COP variability, was adopted as the representative index. In addition, to confirm variability in jump height, jump height was calculated based on flight time obtained from the force plate according to a previous study, using the following equation [40]:

Jump height = (flight time^2^ × gravitational acceleration)/8

#### 2.5.2. mSEBT Data Processing

The reach distances were measured in triplicate in each of the three directions, and the mean values were calculated. The lower-limb length was measured from the anterior superior iliac spine to the medial malleolus. The mean reach distances were then normalized by dividing them by this length, accounting for variation in lower-limb size among participants. This method allows for a relative assessment of dynamic balance ability [13,41]. Additionally, the same measurement procedure was used for all participants to ensure consistency.

#### 2.5.3. WBLT Data Processing

HWD was measured three times, and the average value was calculated. Lower-limb length was determined using the same method as the mSEBT and following the same procedure for all participants. The mean HWD was then normalized by dividing it by the lower-limb length. This normalization aimed to evaluate ankle mobility while accounting for the effect of lower-limb length.

### 2.6. Statistical Analysis

Exploratory factor analysis was conducted to extract the latent factors underlying each variable. Since this study was an exploratory pilot, an a priori sample size calculation was not performed. For factor analysis, a participant-to-variable ratio of about 3–20 or higher is generally recommended [42]. With five variables in this analysis, the ratio was 6.8. While this falls within the suggested range, it is smaller than typical ratios in large-scale studies, so the factor analysis should be viewed as exploratory. The principal axis factor was used for factor extraction. The dynamic postural control-related indicators examined in this study could be inter-related rather than mutually independent. Therefore, oblique rotation (promax rotation) was applied. Promax rotation allows correlations among factors while yielding a simple factor structure, and it is widely used in latent structure analyses in psychology and motor control research [43]. The number of factors was determined through a thorough evaluation using the eigenvalues-greater-than-one criterion (Kaiser criterion) [44], inspection of the scree plot, and parallel analysis. Results showed a two-factor structure based on the Kaiser criterion and scree plot, while parallel analysis indicated a one-factor structure. Since this study was an exploratory pilot examining the theoretical framework of the spatial and temporal aspects of dynamic postural control, a two-factor solution was chosen as a hypothesis-generating approach. This decision considered not only statistical criteria but also theoretical validity and interpretability. Additionally, a factor loading of 0.50 or higher was used as the threshold for substantial contribution to a factor, aligning with previous research that deems loadings of this magnitude practically meaningful [45]. We conducted the Kaiser–Meyer–Olkin (KMO) test to assess the data’s suitability for factor analysis. Statistical analyses were performed using IBM SPSS Statistics version 29.0 (IBM Corp., Armonk, NY, USA). The significance level was set at 5%.

## 3. Results

### 3.1. SDA, mSEBT, and WBLT

The jump height during the vertical jump landing test was 13.3 ± 4.3 cm (mean ± SD).

Table 2 shows the results for the SDA-derived variable (CP), the mSEBT variables (reach distances for the ANT, PM, and PL directions), and the WBLT-derived variable (HWD).

### 3.2. Factor Analysis

According to the established KMO criteria, KMO values of ≥0.90, 0.80–0.89, 0.70–0.79, 0.60–0.69, 0.50–0.59, and <0.50 are considered marvelous, meritorious, middling, mediocre, miserable, and unacceptable, respectively [46]. Therefore, the sampling adequacy in this study was considered mediocre. Further, Bartlett’s test of sphericity had significant results (*p* < 0.001), indicating that the correlation matrix was not an identity matrix. Table 3 displays the correlation coefficients and confidence intervals among the variables, while Table 4 shows the factor loadings and communalities for each variable. Table 5 provides the factor correlation matrix. The factor analysis revealed that the two-factor solution explained 79.77% of the variance, with factor 1 accounting for 47.24% and factor 2 for 32.53%. Variables with factor loadings of ≥0.5 on factor 1 were the reach distances for the ANT, PL, and PM directions, whereas CP and HWD had factor loadings of ≥0.5 on factor 2.

## 4. Discussion

In this study, factor analysis was conducted to explore the latent structure among variables related to dynamic postural control assessed by the SDA and mSEBT, and joint function assessed by the WBLT, in young female volleyball players. As a result, two factors were identified: factor 1 comprised indicators associated with spatial dynamic balance ability, and factor 2 included indicators associated with temporal COP variability and ankle mobility. While these results suggest potentially consistent relationships among the SDA, mSEBT, and WBLT, the study’s KMO value of 0.63 indicates only a mediocre sampling adequacy. This suggests that the factor structure may be affected by sample characteristics and that the stability and generalizability of these factors are limited. Therefore, the discussion should be viewed as exploratory and aimed at generating hypotheses.

### 4.1. Factor 1: Spatial Dynamic Balance Ability

In factor 1, the mSEBT components ANT, PL, and PM exhibited high factor loadings. In particular, PL and PM had high values (≥0.9), suggesting that this factor may be associated with spatial dynamic balance ability centered on lower-limb reach performance in the mSEBT. The mSEBT is a task that requires participants to reach as far as possible with the non-support leg while maintaining posture on the support leg, requiring the ability to spatially move the body while controlling the center of gravity within the base of support [14,15]. In particular, PM and PL require coordinated control of the hip abductor muscles, trunk muscles, and muscles surrounding the ankle joint [16,47]. ANT, despite having a factor loading of 0.50 or higher, exhibited a lower loading compared to PL and PM. Additionally, cross-loading was detected, indicating that its contribution to this factor might be more restricted than the other directions. Further, the significant positive correlation between ANT and HWD (r = 0.42, *p* = 0.01) suggests that characteristics of ankle dorsiflexion range of motion may be partially related to ANT performance. These findings indicate that, despite the mSEBT’s common use in evaluating spatial dynamic balance, different reach directions might not reflect a single, unified postural control mechanism. Instead, each direction could be linked to separate aspects of spatial postural regulation.

### 4.2. Factor 2: COP Fluctuation Patterns and Ankle Mobility

For factor 2, the WBLT showed a strong positive factor loading, while CP had a negative loading. These results suggest that this factor relates to the ankle dorsiflexion range of motion and the temporal features of COP variability measured by SDA. In SDA, CP is identified as the point where the short- and long-term areas intersect on the stabilogram diffusion curve. It is also linked to the temporal transition features of COP fluctuation patterns [33]. Conventionally, in SDA, a short CP indicates an earlier transition to sensory feedback control. Meanwhile, a long CP reflects a neural control strategy characterized by prolonged dependence on open-loop control [33]. In this context, previous studies have reported that neurophysiological indicators, such as electroencephalography and electromyography, can be combined with COP time series data to analyze the neurological mechanisms underlying postural control in detail [48]. By contrast, some reports have shown that SDA is a statistical analysis of COP time-series data and does not directly evaluate neural activity itself. Consequently, a unified consensus on its interpretation has not yet been reached [49]. Therefore, when interpreting CP in this study, there is insufficient evidence to consider it as a direct indicator of neural control strategies. Rather, it should be interpreted with caution as an indicator of the temporal characteristics of COP variability.

In this study, although CP showed a negative factor loading, WBLT showed a positive one. This indicates a potential link between ankle dorsiflexion mobility and the timing features of COP variability. Nevertheless, due to the lack of a definitive consensus on how to interpret CP physiologically, the underlying mechanisms remain uncertain. Future research involving neurophysiological assessments is necessary to understand better the connection between ankle mobility and the temporal aspects of COP variability.

In contrast, in this study, the PL and PM components of the mSEBT had low loadings on factor 2. This finding suggests that the spatial reach ability assessed by the mSEBT and the temporal characteristics of COP variability assessed by the SDA do not necessarily reflect the same underlying structure. This study was an exploratory pilot with a small sample size and few variables, so we cannot confirm that these two constructs are independent. Accordingly, the results should be viewed as preliminary evidence indicating that the spatial and temporal aspects of dynamic postural control might represent separate latent structures.

### 4.3. Limitation

This study has several limitations. First, the participants were limited to female volleyball players, who represent a relatively homogeneous population in terms of age, sex, and sport-specific characteristics. Therefore, whether the findings of this study can be generalized to athletes from other sports, sexes, or competitive levels remains unclear.

Second, the CP, mSEBT, and WBLT used in this study do not fully capture the overall latent structure of dynamic postural control; they reflect only a limited aspect, based on partial relationships among these variables. Third, participants were screened via a pre-study questionnaire for a history of CAI and recurrent ankle sprains, and none met these criteria. However, a past single ankle sprain was not an exclusion criterion, so its potential effect cannot be completely dismissed. Fourth, jump height was not standardized before testing, which could have affected COP dynamics during landing. Future research should control jump height to clarify this. Fifth, as a pilot study with a small sample size, the KMO value was 0.63, indicating limited sampling adequacy. Past research shows small samples can skew the number of factors and loadings based on sample traits, possibly leading to different results in other populations [50]. Since only five variables and a limited number of participants were involved, the factor structure might be sample-dependent. Parallel analysis suggested a single-factor model, so the two-factor structure used here should be interpreted cautiously. Overall, these findings should be viewed as hypothesis-generating. Therefore, additional research with larger sample sizes is necessary to assess the consistency and reliability of the factor structure identified in this study.

## 5. Conclusions

In this study, to examine the latent structure underlying dynamic postural control, factor analysis was conducted on multiple indices obtained from the mSEBT, SDA, and WBLT in young female volleyball players. Two factors were identified: one related to spatial dynamic balance, indicated by high factor loadings across all mSEBT reach directions, and another linked to the temporal aspects of COP variability and ankle mobility, characterized by high loadings for CP and WBLT.

However, because this was an exploratory pilot study with a small sample size, cautious interpretation is warranted when assessing the physiological significance of these factors. Therefore, future investigations using larger and more diverse populations should be conducted to validate the reproducibility and generalizability of the factor structure identified in this study, examine its structural validity through confirmatory factor analysis, and conduct a more comprehensive evaluation that includes additional biomechanical variables.

## Figures and Tables

**Table 1:** Characteristics of the participants.

Age (years)	15.2	±	1.5
Height (cm)	157.7	±	6.2
Weight (kg)	52.8	±	7.9
Duration of athletic career (years)	4.1	±	3.3

Mean ± standard deviation.

**Table 2:** mSEBT, SDA, and WBLT variables.

Variable	Mean ± SD	Skewness	Kurtosis	Shapiro–Wilk W	*p*-Value
SDA					
CP (s)	0.80 ± 0.32	1.063	1.663	0.915	0.011
mSEBT					
ANT (%)	0.99 ± 0.06	0.006	−0.676	0.980	0.776
PL (%)	0.78 ± 0.10	0.796	0.508	0.950	0.125
PM (%)	0.88 ± 0.11	0.324	−0.565	0.969	0.441
WBLT					
HWD (%)	0.16 ± 0.03	−0.297	1.407	0.963	0.290

Mean ± standard deviation. CP: critical point, ANT: anterior, PM: posteromedial, PL: posterolateral, HWD: hallux-to-wall distance.

**Table 3:** Correlation coefficients and confidence intervals for each variable.

Variables	r	*p*-Value	95% CI	
			Lower Limit	Upper Limit
ANT–PL	0.52	0.00	0.21	0.73
ANT–PM	0.44	0.01	0.12	0.68
ANT–CP	−0.10	0.56	−0.43	0.24
ANT–HWD	0.42	0.01	0.10	0.67
PL–PM	0.85	<0.001	0.72	0.92
PL–CP	−0.24	0.18	−0.53	0.11
PL–HWD	0.22	0.22	−0.13	0.52
PM–CP	−0.32	0.07	−0.59	0.02
PM–HWD	0.21	0.23	−0.14	0.51
CP–HWD	−0.28	0.11	−0.57	0.06

r: Pearson’s correlation coefficient; ANT: anterior, PL: posterolateral, PM: posteromedial, CP: critical point, HWD: hallux-to-wall distance. 95% confidence intervals were calculated based on Fisher’s z transformation.

**Table 4:** Factor loadings and communalities of parameters for each factor.

	Factor 1	Factor 2	Communality
ANT (%)	**0.672**	0.489	0.691
PL (%)	**0.949**	0.265	0.971
PM (%)	**0.929**	0.292	0.948
CP (s)	−0.270	**−** **0.684**	0.541
HWD (%)	0.272	**0.874**	0.838
Eigenvalue	2.36	1.63	
Variance explained (%)	47.24	32.53	
Cumulative variance (%)	47.24	79.77	

Factor loadings ≥ |0.50| are shown in bold. ANT: anterior, PL: posterolateral, PM: posteromedial, CP: critical point, HWD: hallux-to-wall distance.

**Table 5:** Factor correlation matrix.

	Factor 1	Factor 2
Factor 1	1.00	0.29
Factor 2	0.29	1.00

## Data Availability

The original data presented in this study are publicly available in the institutional repository of Yamagata Prefectural University of Health Sciences, at https://yachts.repo.nii.ac.jp/records/2000180 (accessed on 23 June 2026).

## References

[1] Harper D.J., McBurnie A.J., Dos’Santos T., Eriksrud O., Evans M., Cohen D.D., Rhodes D., Carling C., Kiely J. (2022). Biomechanical and Neuromuscular Performance Requirements of Horizontal Deceleration: A Review with Implications for Random Intermittent Multi-Directional Sports. Sports Med..

[2] Cherni Y., Jlid M.C., Mehrez H., Shephard R.J., Paillard T., Chelly M.S., Hermassi S. (2019). Eight Weeks of Plyometric Training Improves Ability to Change Direction and Dynamic Postural Control in Female Basketball Players. Front. Physiol..

[3] Borzucka D., Kręcisz K., Kuczyński M. (2020). Postural control in top-level female volleyball players. BMC Sports Sci. Med. Rehabil..

[4] Borzucka D., Kręcisz K., Kuczyński M. (2024). Ground reaction forces better than center of pressure differentiate postural control between young female volleyball players and untrained peers. Sci. Rep..

[5] Ogasawara I., Revankar G.S., Konda S., Matsuo T., Aoyama C., Nakata K. (2023). Individual Variation in Adaptive Ability of the Anticipated Postural Stability During a Dual-Task Single-Leg Landing in Female Athletes. Orthop. J. Sports Med..

[6] Takeuchi S., Anan M. (2026). Impaired postural control following unanticipated single-leg landings in female soccer players with chronic ankle instability. J. Biomech..

[7] Guzmán-Muñoz E., Montalva-Valenzuela F., Garcia-Carrillo E., Castillo-Paredes A., López-Gil J.F., Narrea Vargas J.J., Yáñez-Sepúlveda R., Concha-Cisternas Y. (2026). Postural Balance and Human Movement: An Integrative Framework for Mechanisms, Assessment, and Functional Implications. J. Clin. Med..

[8] Hall K.J., Van Ooteghem K., McIlroy W.E. (2023). Emotional state as a modulator of autonomic and somatic nervous system activity in postural control: A review. Front. Neurol..

[9] Sugimoto D., Myer G.D., Foss K.D.B., Hewett T.E. (2015). Specific exercise effects of preventive neuromuscular training intervention on anterior cruciate ligament injury risk reduction in young females: Meta-analysis and subgroup analysis. Br. J. Sports Med..

[10] Olmsted L.C., Carcia C.R., Hertel J., Shultz S.J. (2002). Efficacy of the Star Excursion Balance Tests in detecting reach deficits in subjects with chronic ankle instability. J. Athl. Train..

[11] Domingues P.C., Serenza F.S., Muniz A.M.S., Fonseca F., Teixeira P.E., de Carvalho R.T. (2018). The relationship between performance on the modified Star Excursion Balance Test and knee muscle strength before and after anterior cruciate ligament reconstruction. Knee.

[12] Clagg S., Paterno M.V., Hewett T.E., Schmitt L.C. (2015). Performance on the Modified Star Excursion Balance Test at the time of return to sport following anterior cruciate ligament reconstruction. J. Orthop. Sports Phys. Ther..

[13] Plisky P.J., Rauh M.J., Kaminski T.W., Underwood F.B. (2006). Star Excursion Balance Test as a predictor of lower extremity injury in high school basketball players. J. Orthop. Sports Phys. Ther..

[14] Gribble P.A., Hertel J., Plisky P. (2012). Using the Star Excursion Balance Test to assess dynamic postural-control deficits and outcomes in lower extremity injury: A literature and systematic review. J. Athl. Train..

[15] Powden C.J., Dodds T.K., Gabriel E.H. (2019). The reliability of the Star Excursion Balance Test and Lower Quarter Y-Balance Test in healthy adults: A systematic review. Int. J. Sports Phys. Ther..

[16] Gabriner M.L., Houston M.N., Kirby J.L., Hoch M.C. (2015). Contributing factors to Star Excursion Balance Test performance in individuals with chronic ankle instability. Gait Posture.

[17] Endo Y., Miura M. (2021). Effects of posture and lower limb muscle strength on the Star Excursion Balance Test in healthy male adults. J. Phys. Ther. Sci..

[18] de la Motte S., Arnold B.L. (2015). Trunk-rotation differences at maximal reach of the Star Excursion Balance Test in participants with chronic ankle instability. J. Athl. Train..

[19] Veronez F., de Oliveira M.R., de Lima-Pardini A.C., Coelho D.B., Costa P.H.L., Teixeira L.A. (2024). The use of nonlinear analysis in understanding postural control: A scoping review. Hum. Mov. Sci..

[20] Zhang X., Schütte K.H., Vanwanseele B., De Clercq D., Vanrenterghem J. (2017). Foot muscle morphology is related to center of pressure sway and control mechanisms during single-leg standing. Gait Posture.

[21] Doyle R.J., Ragan B.G., Rajendran K., Redfern M.S. (2008). Generalizability of stabilogram diffusion analysis of center of pressure measures. Gait Posture.

[22] Fong C.M., Blackburn J.T., Norcross M.F., McGrath M., Padua D.A. (2011). Ankle-dorsiflexion range of motion and landing biomechanics. J. Athl. Train..

[23] Malloy P., Morgan A., Meinerz C., Geiser C., Kipp K. (2015). The association of dorsiflexion flexibility on knee kinematics and kinetics during a drop vertical jump in healthy female athletes. Knee Surg. Sports Traumatol. Arthrosc..

[24] Akbari H., Shimokochi Y., Sheikhi B. (2023). Ankle dorsiflexion range of motion and landing postures during a soccer-specific task. PLoS ONE.

[25] Trajković N., Kozinc Ž., Smajla D., Šarabon N. (2021). Relationship between ankle strength and range of motion and postural stability during single-leg quiet stance in trained athletes. Sci. Rep..

[26] Hogan K.K., Powden C.J., Hoch M.C. (2016). The influence of foot posture on dorsiflexion range of motion and postural control in those with chronic ankle instability. Clin. Biomech..

[27] Horak F.B. (2006). Postural orientation and equilibrium: What do we need to know about neural control of balance to prevent falls?. Age Ageing.

[28] Jasimi Zindashti N., Noamani A., Vette A.H., Rouhani H. (2025). A narrative review on dynamic postural stability and neuromuscular control of balance. Trans. Can. Soc. Mech. Eng..

[29] Vicenzino B., Branjerdporn M., Teys P., Jordan K. (2006). Initial changes in posterior talar glide and dorsiflexion of the ankle after mobilization with movement in individuals with recurrent ankle sprain. J. Orthop. Sports Phys. Ther..

[30] Onofrei R.R., Amaricai E., Petroman R., Suciu O. (2019). Relative and absolute within-session reliability of the modified Star Excursion Balance Test in healthy elite athletes. PeerJ.

[31] Zhang Y., Pei S., Martin R.L. (2026). A Systematic Review of the Star Excursion Balance Test to Define Clinically Meaningful Psychometric Values. Sports Health.

[32] Bennell K.L., Talbot R.C., Wajswelner H., Techovanich W., Kelly D.H., Hall A.J. (1998). Intra-rater and inter-rater reliability of a weight-bearing lunge measure of ankle dorsiflexion. Aust. J. Physiother..

[33] Collins J.J., De Luca C.J. (1993). Open-loop and closed-loop control of posture: A random-walk analysis of center-of-pressure trajectories. Exp. Brain Res..

[34] Vuillerme N., Vincent H. (2006). How performing a mental arithmetic task modify the regulation of centre of foot pressure displacements during bipedal quiet standing. Exp. Brain Res..

[35] Peters M. (1988). Footedness: Asymmetries in foot preference and skill and neuropsychological assessment of foot movement. Psychol. Bull..

[36] Afonso J., Ramirez-Campillo R., Lima R.F., Laporta L., Paulo A., Castro H.d.O., Costa G.D.C.T., García-De-Alcaraz A., Araújo R., Silva A.F. (2021). Unilateral versus Bilateral Landing after Spike Jumps in Male and Female Volleyball: A Systematic Review. Symmetry.

[37] Elias L.J., Bryden M.P., Bulman-Fleming M.B. (1998). Footedness is a better predictor than is handedness of emotional lateralization. Neuropsychologia.

[38] van Melick N., Meddeler B.M., Hoogeboom T.J., Nijhuis-van der Sanden M.W.G., van Cingel R.E.H. (2017). How to determine leg dominance: The agreement between self-reported and observed performance in healthy adults. PLoS ONE.

[39] Rizzato A., Benazzato M., Cognolato M., Grigoletto D., Paoli A., Marcolin G. (2023). Different neuromuscular control mechanisms regulate static and dynamic balance: A center-of-pressure analysis in young adults. Hum. Mov. Sci..

[40] Flanagan E.P., Comyns T.M. (2008). The Use of Contact Time and the Reactive Strength Index to Optimize Fast Stretch-Shortening Cycle Training. Strength Cond. J..

[41] Gribble P.A., Hertel J. (2003). Considerations for Normalizing Measures of the Star Excursion Balance Test. Meas. Phys. Educ. Exerc. Sci..

[42] Mundfrom D.J., Shaw D.G., Ke T.L. (2005). Minimum Sample Size Recommendations for Conducting Factor Analyses. Int. J. Test..

[43] Watkins M.W. (2018). Exploratory Factor Analysis: A Guide to Best Practice. J. Black Psychol..

[44] Kaiser H.F. (1960). The Application of Electronic Computers to Factor Analysis. Educ. Psychol. Meas..

[45] Hair J.F., Black W.C., Babin B.J., Anderson R.E. (2019). Multivariate Data Analysis.

[46] Kaiser H.F. (1974). An Index of Factorial Simplicity. Psychometrika.

[47] Pinheiro L.S.P., Ocarino J.M., Bittencourt N.F.N., Souza T.R., Martins S.C., Bomtempo R.A.B., Resende R.A. (2020). Lower limb kinematics and hip extensor strength are associated with performance of runners at high risk of injury during the modified Star Excursion Balance Test. Braz. J. Phys. Ther..

[48] Hua T., Zhang Y., Wang J., Li X., Chen Z., Liu H. (2024). Nonlinear dynamics of postural control system under visual-vestibular habituation balance practice: Evidence from EEG, EMG and center of pressure signals. Neurosci. Lett..

[49] Peterka R.J. (2000). Postural control model interpretation of stabilogram diffusion analysis. Biol. Cybern..

[50] Costello A.B., Osborne J.W. (2005). Best practices in exploratory factor analysis: Four recommendations for getting the most from your analysis. Pract. Assess. Res. Eval..

